# Antioxidant Activities of Celery and Parsley Juices in Rats Treated with Doxorubicin 

**DOI:** 10.3390/molecules15096193

**Published:** 2010-09-03

**Authors:** Jovanka Kolarovic, Mira Popovic, Janka Zlinská, Svetlana Trivic, Matilda Vojnovic

**Affiliations:** 1 Institute for Child and Youth Health Care of Vojvodina, Department of Hematology/Oncology, Novi Sad, Serbia, E-Mail: jovanka.kolarovic@gmail.com (J.K.); 2 Department of Chemistry, Faculty of Science, University of Novi Sad, Serbia; 3 University of Central Europe in Skalica, Slovakia; 4 The Health Center, 21000 Novi Sad, Serbia

**Keywords:** celery and parsley juices, doxorubicin, reduced glutathione, FRAP, Cyt P450

## Abstract

We have examined the influence of diluted pure celery and parsley leaf and root juices and their combinations with doxorubicin on the antioxidant status [as measured by the content of reduced glutathione (GSH) and ferric reducing antioxidant power (FRAP)] in liver homogenate and hemolysate and on the contents of cytochrome P450 in liver homogenate. It was found that doxorubicin significantly decreased the content of reduced glutathione and the total antioxidative status (FRAP) in liver homogenate and hemolysate, while celery and parsley juices alone and in combination with doxorubicin had different actions. Doxorubicin and celery juice had no effect on content of cytochrome P450. However, in combination with doxorubicin, parsley root juice significant increased, and parsley leaves juice decreased the cytochrome P450 content (compared to doxorubicin treated animals). Only parsley root juice significantly increased the content of cytochrome P450.

## 1. Introduction

Doxorubicin is among the most effective and widely used cytotoxic drugs from the anthracycline group. It is used in treatment of acute leukemias, lymphomas and different types of solid tumors such as breast, liver and lung cancers. Doxorubicin is significantly toxic to most tissues, organs and systems of organs. It is extremely toxic to the heart and can cause permanent damage and even death. There is increasing evidence that essential components of myocardial energy metabolism are among the highly sensitive and early targets of doxorubicin-induced damage [1,2].

It is thought that doxorubicin causes antioxidative stress by inducing free oxygen radicals that lead to lipid peroxidation in mitochondrial membranes and the sarcoplasmatic reticulum, thus damaging cardiac muscle. Different authors have tried to combine potentially cardiotoxic drugs with extracts of medical herbs used as herbal remedy, in an attempt to reduce their side effects [3,4], as antioxidants from natural sources may be useful in the protection of doxorubicin induced cardiotoxicity [4,5].

Parsley (*Petroselinum hortense Hoffm. 1814;* the earlier botanical name *Petroselinum crispum* [Mill.] Nyman ex A. W. Hill, Apiaceae; in 1764. Linnaeus classified it *Apium petroselinum* L.) contains flavonoids (apiin and luteolin) and essential oil (apiol and myristicin), responsible for both the medical uses and toxicity of parsley. Furanocoumarins (psoralen, bergapten, isoimperatorin, oxypeucedanin, xanthoxin, trioxalen and angelicin) are other important chemical constituents of parsley plants [6,7].

In folk medicine parsley is used for menstrual disorder ailments, as an emmenagogue, galactagogue and stomachic, and externally against head lice. Results of numerous investigations point out to the antioxidant properties of parsley. For example, the flavonoid apigenin, one of the components of parsley plant, was shown to express strong antioxidant effects by increasing the activities of antioxidant enzymes and related to that, decreasing the oxidative damage to tissues. Potential for anticancer activity by parsley was reported as well [7,8].

Celery *(Apium graveolens* L. Sp. Pl. 264 (*1753*), Apiaceae) is a medicinal herb used as a food, and also in traditional medicine. It contains aromatic substances in the roots, stem and leaves. The healing properties of celery are due to the essential oil and flavonoids, mostly apiin and apigenin. Celery contains an essential oil (d-limonene and selinene, santalol, eudesmol, apiol myristicin, *etc*) [7,9].

The essential oil from celery exhibits antifungal and antibacterial actions. Celery can lower blood pressure and regulate heart function. Celery can be used to slow down and treat complications caused by diabetes because it influences the blood glucose level by stimulating the pancreas to insulin secretion [7].

It is well known that celery can cause photodermatitis and contact dermatitis and act as aphrodisiac.It can cause sedation and irritation and be responsible for spasmolytic action. A number of chemical compounds present in celery seeds show antiinflammatory and analgesic effects. Apigenin from celery seed exhibits an antiaggregation effect *in vitro*. It is reported to inhibit contractions of the isolated smooth muscle of the thoracic aorta [7].

Some flavonoids have mutagenic and/or prooxidant effects. Cytochromes P450 (CYPs), monooxygenases metabolizing xenobiotics (e.g. drugs, carcinogens) interact with flavonoids and other endogenous substrates. Flavonoids induce the expression of several CYPs and modulate (inhibit or stimulate) their metabolic activity. Some CYPs participate in metabolism of flavonoids. If the flavonoids are coadministered with other drugs, careful attention should be paid to the metabolism because of possible interactions [10].

Numerous studies show that active principles from plants have multiple effects on metabolism and may alter activity of different drugs. Secondary biomolecules from plants might have diverse effects such as: antibacterial and antiviral activity, anti-inflammatory, anti-angionic, analgesic and antiallergic effects, hepatoprotective, cytotoxic, apoptotic, estrogenic and antiestrogenic properties, antioxidant and prooxidant effects, *etc.* [11,12,13].

Our previous results have pointed at antioxidant and hepatoprotective effects of celery and parsley extracts, as well as influence on pharmacodynamic activities [7]. These findings have led us to examine the influence of celery and parsley root and leaf juices on total antioxidant status and content of reduced glutathione in liver homogenate and hemolysate in doxorubicin treated rats. We also investigated the effect of treatment with doxorubicin (alone and in combination with celery and parsley roots and leaves juices) on the cytochrome P450 content.

## 2. Results and Discussion

**Figure 1:**
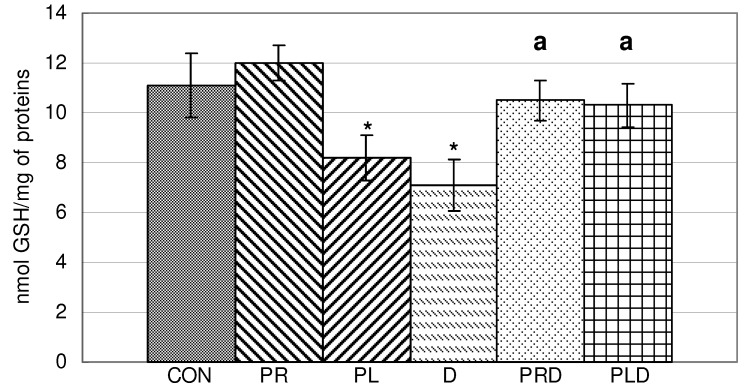
Content of reduced glutathione in liver homogenate of animals treated with doxorubicin, parsley leaves and roots juices and their combination. t-test, n = 6; compared to CON group: * p < 0.05, compared to D group: ^a^ p < 0.05.

In liver homogenate the content of reduced glutathione was significantly decreased by parsley leaf juices and doxorubicin (Figure 1). Parsley root and leaf juices and their combination with doxorubicin caused a significant increase in GSH content compared to doxorubicin alone. Since the content of reduced GSH is the important indicator of antioxidative status, we can conclude that positive synergism was observed. 

**Figure 2:**
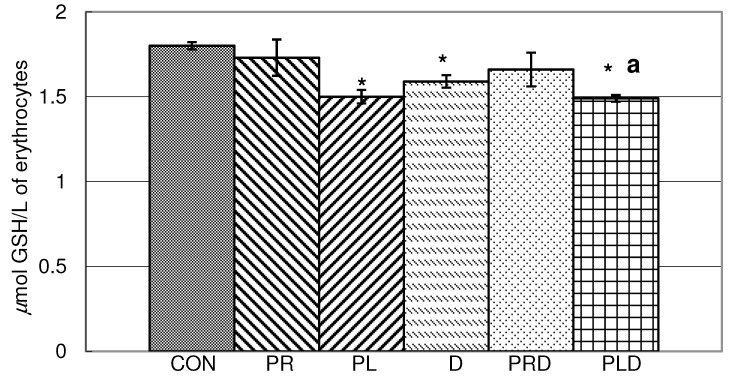
Content of reduced glutathione in blood hemolysate of animals treated doxorubicin, parsley leaves and roots juices and their combination. t-test, n = 6; compared to CON group: * p <0.05, compared to D group: ^a^ p <0.05.

In blood hemolysate reduced glutathione content was significantly decreased by parsley leaf juice, doxorubicin and by a combination of doxorubicin and parsley leaf juice (Figure 2). Parsley leaf juice in combination with doxorubicin caused a reduction in GSH content compared to doxorubicin alone.

In liver homogenate the content of reduced glutathione was significantly increased by celery root and leaf juices and decreased by doxorubicin and combinations of doxorubicin and celery root juice. In blood hemolysate reduced glutathione content was significantly decreased only by doxorubicin [3]. As expected, doxorubicin reduced the GSH content in liver homogenate and blood hemolysate. 

In our previous research, celery and parsley have exhibited protective effects in liver homogenate and in blood hemolysate of experimental animals treated with doxorubicin or CCl_4_ [3,14]. The growth inhibitory activity of doxorubicin or cisplatin, as a single agent, may be modified by combinations of *Phyllanthus emblica* or *Terminalia bellerica* extracts and be synergistically enhanced in some cases [15].

Prevention of doxorubicin induced oxidative stress damage of rat heart by sesame oil, supports the hypothesis that at least part of the mechanism of cardiotoxicity could be attributed to the overproduction of free radicals. Oxidative damage to heart contributes to the myocardial toxicity induced by doxorubicin in male rats. These effects might be limited by the use of sesame oil whose protective effects may be due to its antioxidant properties [16].

Beyond this, many investigators have proposed that oxidative stress is an important component of the toxicity caused by most chemicals that are activated to electrophiles. Electrophiles also deplete reduced glutathione, one of the defenses against damage from reactive oxygen species such as hydroperoxides. Even more subtle changes in the redox balance of a cell may be critical, in that many transcription factors and other signaling systems are controlled by redox events [17].

**Figure 3:**
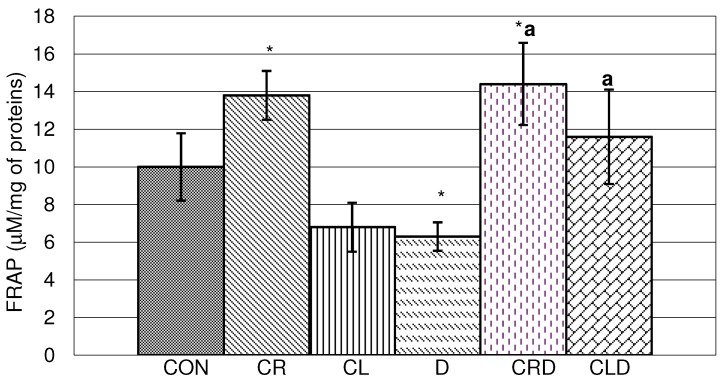
Effects of doxorubicin, celery leaf and root juices and their combination on FRAP antioxidant capacities in liver homogenate. t-test, n = 6; compared to CON group:* p < 0.05, compared to D group: ^a^ p < 0.05.

Figure 3 presents the antioxidative status of liver homogenate of experimental animals. As expected, celery root juice significantly increased the antioxidative capacity compared to control, while doxorubicin reduced it. In combination of doxorubicin and celery root and leaf juices, positive synergism was observed and the antioxidative capacity was significantly higher. Recently, much attention has been focused on the protective effects of antioxidants and naturally occurring substances against doxorubicin -induced cardiotoxicity [16].

In a paper by Sugiyama *et al*. it was shown that patients who had drank green tea or theanine was added to their food exhibited less severe side effects from doxorubicin treatments [18]. Production of antibacterial proteins in the blood was up to five times higher in the tea-drinkers, an indicator of a stronger immune response [19].

**Figure 4:**
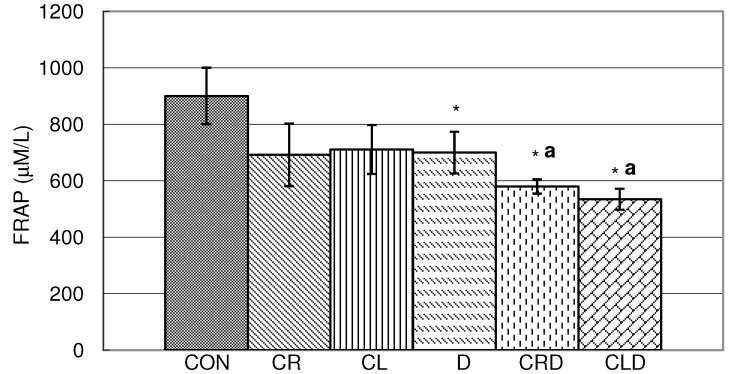
Effects of doxorubicin, celery leaf and root juices and their combination on FRAP antioxidant capacities in hemolysate. t-test, n = 6; compared to CON group:* p < 0.05, compared to D group: ^a^ p < 0.05.

In hemolysate, doxorubicin and combination of doxorubicin and both celery juices (leaves and roots) reduced antioxidative capacity compared to the control (CON) (Figure 4). It is interesting to note that combinations of celery roots and leaves juices with doxorubicin (CRD and CLD) significantly reduced FRAP when compared to doxorubicin. This could be explained by presence of prooxidant flavonoids with a phenolic B ring, especially those with one ring [20], although they primarily act as antioxidants [21].

**Figure 5:**
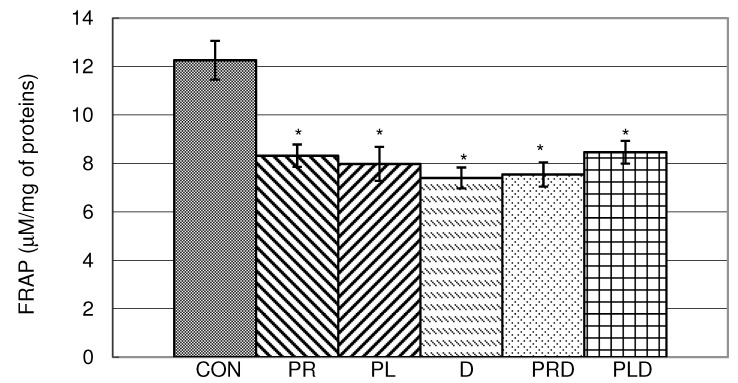
Effects of doxorubicin, parsley leaf and root juices and their combination on FRAP antioxidant capacities in liver homogenate. t-test, n = 6; compared to CON group:* p < 0.05, compared to D group: ^a^ p < 0.05.

In Figure 5 it is obvious that all parsley and celery juices, doxorubicin and their combination significantly reduced FRAP in liver homogenate. It is interesting that parsley juices increase antioxidative capacity [21], although some references indicate that extracts of parsley have prooxidative properties [22]. Doxorubicin as a cytotoxic drug causes reduction of antioxidative capacity with similar FRAP values as parsley juices. The combination of the medicine and parsley juices did not show positive synergism (Figure 5).

**Figure 6:**
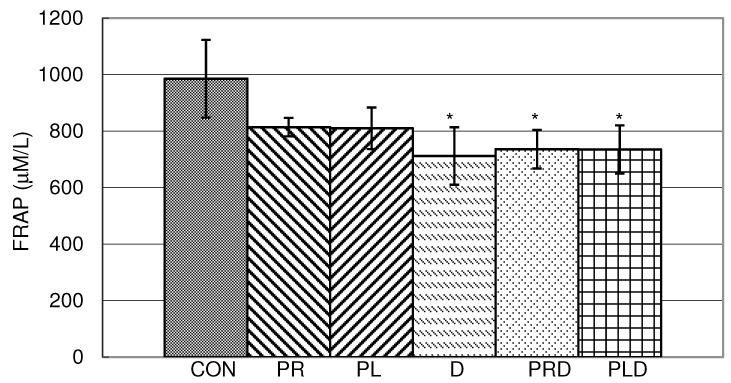
Effects of doxorubicin, parsley leaf and root juices and their combination on FRAP antioxidant capacities in hemolysate. t-test, n = 6; compared to CON group:* p < 0.05, compared to D group: ^a^ p < 0.05.

Results obtained by determination of total antioxidative status in liver homogenate were very similar to the results of investigation of antioxidative status in blood hemolysate of rats treated with parsley juices, doxorubicin and their combinations (Figure 6).

**Figure 7:**
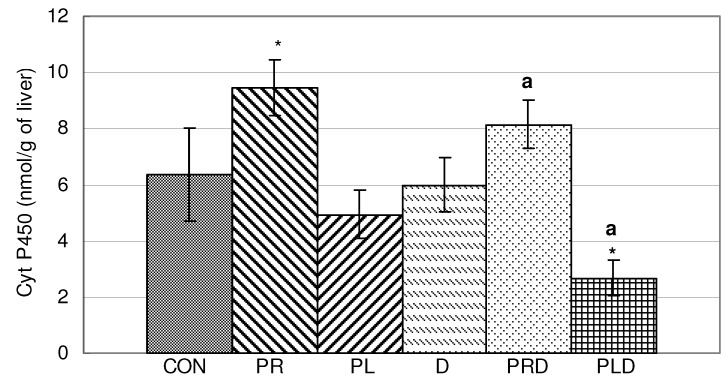
Effects of doxorubicin, parsley leaves and roots juices and their combination on Cyt P450 in liver homogenate. t-test, n = 6; compared to CON group: * p < 0.05, compared to D group: ^a^ p < 0.05.

Parsley root juice (PR) significantly increased the content of Cyt P450, while the combination of parsley leaf juice with doxorubicin (PLD) significantly decreased it in liver homogenate when compared to untreated animals (CON). Treatments with doxorubicin, parsley leaf juice and their combination significantly reduced the content of Cyt P450, while treatments with root juice significantly increased it (Figure 7).

**Figure 8:**
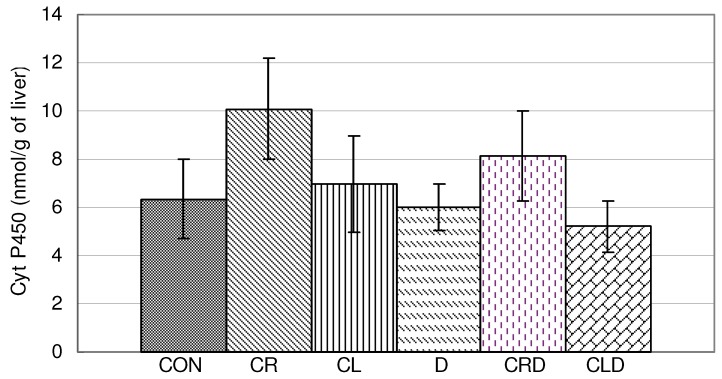
Effects of doxorubicin, celery leaves and roots juices and their combination on Cyt P450 in liver homogenate. t-test, n = 6; compared to CON group: * p < 0.05, compared to D group: ^a^ p < 0.05.

Celery root and leaf juices applied alone and in combination with doxorubicin did not change the content of Cyt P450 in liver homogenate when compared to the control (Figure 8).

A number of naturally occurring flavonoids have been shown to modulate the CYP450 system, including the induction or inhibition of these enzymes [10]. Based on determined protein levels and specific enzyme activities, isoquercitrin, (a constituent of celery plants) was the most efficient inducer of CYP1A1 and CYP1A2 in rat liver. On the other hand, the aglycone quercetin had no effect on the protein levels, but caused a concentration-dependent increase of CYP1A1 mRNA in MCF7 cells [23]. 

In normal metabolism Cyt P450 produce H_2_O_2_ and O_2_^−^. Formation of reactive oxygen species (ROS) can be induced or inhibited with various xenobiotics. The rates of oxygen reduction are very fast *in vitro* but could easily be measured. The question can be raised as to whether these are equally fast *in vivo* [17].

## 3. Experimental

### 3.1. Plant Material

Whole plants of cultivated *Petroselinum hortense* Hoffm. 1814*.*, and *Apium graveolens* L were collected in Juny 2009 at Veternik (Vojvodina province), Republic of Serbia. The voucher specimens *Petroselinum hortense* Hoffm. 1814, No 2-1797, Serbia, Novi Sad, Veternik (UTM 34T DR 2 01), 26.06.2009., det.: Goran Anačkov and *Apium graveolens* L. 1753, No 2-1798, Serbia, Novi Sad, Veternik (UTM 34T DR 2 01), 26.06.2009., det.: Goran Anačkov were confirmed and deposited at the Herbarium of the Department of Biology and Ecology (BUNS Herbarium), Faculty of Natural Sciences, University of Novi Sad.

Whole fresh plants were used in this study. Juices of celery and parsley leaves and roots were prepared identically. Leaves were separated from roots. Fresh leaves were ground in a blender and5% solution (v/v) was prepared by diluting pure juice with distilled water. The same blender was used for grounding roots and a 5% (v/v) solution was prepared by diluting with distilled water.

### 3.2. Animal Treatment

This investigation was conducted on sexually mature male Whistar laboratory rats, with an average body weight of 250-300 grams and ages up to 3 months. Animal care and all experimental procedures were conducted in accordance with the Guide for the Care and Use of Laboratory Animal Resources, edited by Commission of Life Sciences, National Research Council, Male and female Hanover National Medical Institute (Hann NMRI). Rats were bred in the vivarium at the Department of Pharmacology, Toxicology and Clinical Pharmacology, Medical Faculty, University of Novi Sad, Serbia. Animals were kept in standard plexiglass cages at constant room temperature 21 ± 1ºC and humidity 55% ± 1.5%, with circadian rhythm (day/night). They were fed standard laboratory rat feed, produced by the Veterinary Institute in Zemun. Animals were given free access to food and fluid (water or fresh celery or parsley root or leaf juices). The average dose of doxorubicin was selected on the basis of common human dosages and Clark's formula.

In the first part of the experiments animals were treated with doxorubicin, celery root and leaf juices and their combination. Experimental animals were divided into six experimental groups, each comprising six animals. Animals from the control group (CON) only drank water *ad libitum*; animals from group CR drank only celery root juice instead of water; group CL was given only celery leaf juice; animals from group CRD exclusively drank celery root juice and received doxorubicin (1.5 mg/kg) intraperitoneally (i.p.) four times in 14 days (on days 1, 5, 9, 13); group CLD drank only celery leaf juice and was treated with doxorubicin (1.5 mg/kg i.p.) on days 1, 5, 9, 13; group D drank water and received doxorubicin (1.5 mg/kg i.p.) on days 1, 5, 9, 13. In the second part of the experiment the same procedure was repeated with parsley root and leaf juices instead of celery juices. Animals from the control group (CON) drank only water *ad libitum*; animals from group PR drank only parsley root juice instead of water; group PL was given only parsley leaf juice; animals from group PRD exclusively drank parsley root juice and received doxorubicin (1.5 mg/kg) intraperitoneally (i.p.) four times in 14 days (on days 1, 5, 9, 13); group (PLD) drank only parsley leaf juice and was treated with doxorubicin (1.5 mg/kg i.p.) on days 1, 5, 9, 13; group (D) drank water and received doxorubicin (1.5 mg/kg i.p.) on days 1, 5, 9, 13. Non-doxorubicin treated groups received 0.9% sodium chloride (1 mL/kg body weight) intraperitoneally every four days instead of doxorubicin. Intervals between two concomitant applications of doxorubicin resemble the most frequent treatment schedule used in human medicine. On the day 17 (3 days after the last dose of doxorubicin) all animals were sacrificed under urethane anesthesia. Blood samples were collected and livers were removed and homogenized.

### 3.3. Biochemical Assays

Total antioxidant status was measured in blood hemolysate and liver homogenate. Liver was homogenized in a Potter homogenizer with 50 mM TRIS-HCl, 250 mM sucrose in ratio 1:3 at 4 ^o^C. Obtained homogenate was filtered. The content of reduced glutathione (GSH) was determined in blood after Beuthler *et al.* [24] and in the liver after Kapetanovic and Mieyal [25]. The total protein content in liver was determined after Gornall *et al*. [26]. Total content of cytochome P-450 in rat liver homogenate was determined after Matsubara *et al.* [27].

The *ferric reducing antioxidant power* (FRAP) assay [28] measures antioxidant power with the help of a redox reaction, whereby ferric (Fe^3+^) to ferrous ion reduction at low pH causes the formation of a coloured ferrous–tripyridyltriazine complex. FRAP values are obtained by comparing the absorbance change at 593 nm in test reaction mixtures with those containing ferrous ions in known concentration. In the FRAP assay, reductants (‘antioxidants’) in the sample reduce the Fe(III)*/*tripyridyltriazine complex, present in stoichiometric excess, to the blue ferrous form, with an increase in absorbance at 593 nm. Ethanolic solutions of known FeSO_4_ concentration, in the range of 20-500 μM, were used for obtaining the calibration curve. The FRAP value was defined as the concentration of antioxidant having a ferric reducing ability equivalent to that of 1 μM FeSO_4_. The change in absorbance is proportional to the combined (total) ferric reducing*/*antioxidant power (FRAP value) of the antioxidants in the sample. 

### 3.4. Chemicals

2,4,6-Tripyridyl-*s*-triazine (TPTZ) was obtained from BDH Chemicals Ltd (Poole, England).Doxorubicin was obtained from Pharmacia & Upjohn Inc (New Haven, CT, USA). 5,5'-Dithiobis (2-nitrobenzoic acid (DTNB) was obtained from Merck (Darmstadt, Germany). All chemicals used were of analytical grade.

### 3.5. Statistical Analysis

Results of biochemical analyses are presented as the mean value ± standard deviation (SD). The difference between control and test groups was analyzed using the Student t-test (significant difference at p ≤ 0.05 confidence level). 

## 4. Conclusions

On the basis of the obtained results we can conclude that doxorubicin decreased the content of reduced glutathione and antioxidative status in blood hemolysate and liver homogenate but did not have any effect on the content of cytochrome P450. 

Celery root juice increased antioxidative capacity *i.e.* reduced glutathione content [3] and the total antioxidative capacity (FRAP) in liver homogenate. Celery leaf juice increased GSH content [3], but did not influence FRAP in liver homogenate.

Celery root and leaf juices in combination with doxorubicin had protective effects, *i.e.* they increased the total antioxidative capacity (FRAP) in liver homogenate compared to animals treated with doxorubicin alone. 

Parsley root and leaf juices in combination with doxorubicin increased the content of reduced glutathione in liver homogenate compared to doxorubicin treatment, suggesting possible protective effects.

All other treatments with celery and parsley juices, alone or in combination with doxorubicin reduced the total antioxidative status in both blood hemolysate and in liver homogenate. This could be explained by the fact that flavonoids and other classes of plant antioxidants can act as prooxidants under certain conditions. Further research using other more precise methods for determination of total antioxidative activity is needed to explain the obtained results.

Recent investigations have shown that content of cytochrome P450 can be used as a marker of oxidative stress. Parsley roots and leaves juice in combination with doxorubicin had opposite effect on the content of Cyt P450 compared to doxorubicin. 

*In vivo* investigations are very complex. Results are not easily explained since some components of parsley and celery juices act as antioxidants, but also as prooxidants. Our further investigation is aimed towards explanation of effects of celery and parsley juices on antioxidant systems.
